# *Candida albicans*: A Comprehensive View of the Proteome

**DOI:** 10.1021/acs.jproteome.4c01020

**Published:** 2025-03-14

**Authors:** Leticia Gomez-Artiguez, Samuel de la Cámara-Fuentes, Zhi Sun, María Luisa Hernáez, Ana Borrajo, Aída Pitarch, Gloria Molero, Lucía Monteoliva, Robert L. Moritz, Eric W. Deutsch, Concha Gil

**Affiliations:** † Microbiology and Parasitology Department, Faculty of Pharmacy, 16734 Complutense University of Madrid, 28040 Madrid, Spain; ‡ Proteomics Unit, Faculty of Pharmacy, Complutense University of Madrid, 28040 Madrid, Spain; § 7268 Institute for Systems Biology, 401 Terry Ave North, Seattle, Washington 98109, United States

**Keywords:** *Candida albicans*, PeptideAtlas, Proteomics, Tutorial, Post-translational modification

## Abstract

We describe a new release of the *Candida albicans* PeptideAtlas proteomics spectral resource (build 2024-03), providing a sequence coverage of 79.5% at the canonical protein level, matched mass spectrometry spectra, and experimental evidence identifying 3382 and 536 phosphorylated serine and threonine sites with false localization rates of 1% and 5.3%, respectively. We provide a tutorial on how to use the PeptideAtlas and associated tools to access this information. The *C. albicans* PeptideAtlas summary web page provides “Build overview”, “PTM coverage”, “Experiment contribution”, and “Data set contribution” information. The protein and peptide information can also be accessed via the *Candida* Genome Database via hyperlinks on each protein page. This allows users to peruse identified peptides, protein coverage, post-translational modifications (PTMs), and experiments that identify each protein. Given the value of understanding the PTM landscape in the sequence of each protein, a more detailed explanation of how to interpret and analyze PTM results is provided in the PeptideAtlas of this important pathogen. *Candida albicans* PeptideAtlas web page: https://db.systemsbiology.net/sbeams/cgi/PeptideAtlas/buildDetails?atlas_build_id=578.

## Graphical abstract

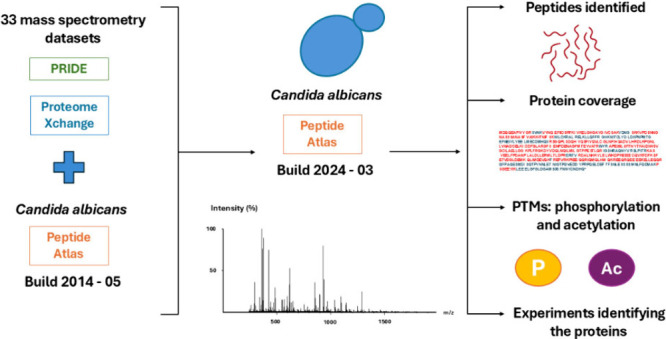

## 1 Introduction

*Candida albicans* is a dimorphic fungus that is a component of commensal human microbiota. However, under certain conditions, it can cause invasive candidiasis, particularly in immunocompromised and critically ill patients.^1,2^ This is one of the primary nosocomial infections, especially among patients in intensive care units, posing significant public health risk.^3,4^ Consequently, the World Health Organization (WHO) has included this organism in its priority list of fungal pathogens.^5^

As a result, the search for diagnostic and prognostic biomarkers of candidiasis has increased, as have efforts to better understand clinically relevant biological processes for intervention. This has led to the development of multiple proteomics studies to understand the proteome complement and identify potential targets of intervention. However, despite the extensive efforts on clinical aspects from the proteomics view, there were no *C. albicans* online public proteomic repositories until the first *C. albicans* PeptideAtlas was published in 2014.^6^ PeptideAtlas is a multiorganism, publicly accessible compendium of mass spectrometry (MS) identified peptides identified from a large set of tandem mass spectrometry proteomics experiments through data reprocessing of MS data sets available through ProteomeXchange^7^ with the Trans-Proteomic Pipeline (TPP)^8^ and makes an integrated view of the results presented back to the community via a consolidated web resource. In spite of being heavily focused on the human proteome since the first publication in 2005 ^9^ PeptideAtlas has also created builds for multiple other species, such as pig (*Sus scrofa*),^10^ chicken (*Gallus gallus*),^11^ plant species (*Arabidopsis thaliana* ^12^ and *Zea mays* ^13^), several yeast species (*Saccharomyces cerevisiae,* ^14^ *Schizosaccharomyces pombe,* ^15^ and *C. albicans* ^6^), and some bacteria species (*Staphylococcus aureus* ^16^ and *Pseudomonas aeruginosa* ^17^), all available under a common interface.

In the 2014 *C. albicans* PeptideAtlas, we provided a significant, large-scale public proteomic resource for the study of this opportunistic pathogenic fungus. It initially cataloged over 2,500 proteins, covering approximately 41% of the predicted proteome. This achievement marked unprecedented proteome coverage for *C. albicans* and distinguished it as the first human fungal pathogen included in the PeptideAtlas project. However, this initial coverage was notably lower than that of other yeast species, such as *S. cerevisiae* (61%)^14^ and *S. pombe* (71%).^15^ Consequently, a subsequent update^18^ expanded the data set to 4,115 identified proteins, thereby increasing the proteome coverage to 66% and bringing it closer to the levels observed in these other yeast species.

Since the last update in 2016, there has been a notable increase in the number of proteomic studies on *C. albicans* (Supplementary Figure S1), making it feasible to undertake a second update of PeptideAtlas. A rigorous search was conducted on Proteomics Identifications Database (PRIDE),^19^ a proteomics repository, selecting studies that adhered to established criteria. These novel data sets, along with previously archived raw MS data files from the two preceding versions of the *C. albicans* PeptideAtlas, have undergone comprehensive reprocessing and analysis through the TPP^8,20^ utilizing an identified peptide sequence database featuring allele-specific sequences from the *Candida* Genome Database (CGD) in conjunction with one from UniProtKB. The outcome is the generation of the most comprehensive *C. albicans* proteomics resource to date, featuring unprecedented proteome coverage and incorporating, as a novelty, the calculation of the probabilities of two post-translational modifications (PTMs) in the amino acid sequence, phosphorylation and acetylation. Considering both the existing and newly incorporated tools, we present a tutorial for utilizing the *C. albicans* PeptideAtlas. Additionally, we provide updates on the process to develop the resource, and the improvements made.

## 2 Materials and Methods

### 2.1 Selection of PRIDE Submissions

ProteomeXchange Data set selections (i.e., PXD records) were defined in PRIDE^19^ according to various criteria: submissions from 2015 onward, studies involving the strain SC5314 widely used in the *C. albicans* field or its derived mutants, proteomic analysis conducted via LC-MS/MS and DDA, absence of protein glycosylation enrichment, and utilization of the CGD for peptide assignment. Thirty-three PXD data sets downloaded met these criteria, and detailed information about them can be found in Supplementary Table S1.

### 2.2 The TPP Data Processing Pipeline

For each PXD, we gathered data information regarding the protease, whether the samples were isotopically labeled, PTM enrichment techniques utilized, and the *Candida* strains selected for the experiments. According to the information collected, the runs of each PXD data set were organized into one or more sub experiments. MS vendor-format raw files were converted to mzML files using ThermoRawFileParser^21^ for Thermo Fisher Scientific instruments, AB_SCIEX_MS_Converter for SCIEX wiff files, and tdf2mzml for Bruker timsTOF raw files. mzML files were searched using MSFragger^22^ and then processed through the TPP^8,20,24^ v6.4.x Pillar, Build 202403120129-9139 for peptide and protein identification. The protein search database included (i) Assembly 22 (A22-s07-m01-r202) (http://www.candidagenome.org/) with 6,226 sequences, (ii) UniProtKB reference proteome UP000000559 ^23^ with 6,036 sequences, (iii) 498 contaminant protein sequences frequently observed in proteome samples, and (iv) a sequence-shuffled decoy counterpart. For the database searching, the following parameters were used: search_enzyme_name_1=trypsin, allowed_missed_cleavage_1=2, and num_enzyme_termini=1. The following variable modifications were set: oxidation of Met or Trp (+15.9949), peptide N-terminal Gln to pyro-Glu (−17.0265), peptide N-terminal Glu to pyro-Glu (−18.0106), deamidation of Asn (+0.9840), protein N-term acetylation (+42.0106), Ser, Thr, Tyr, and Ala (+79.9663) if phosphorylated peptides were enriched, acetylation of Lys (+42.0106) if acetyl-peptides were enriched, hydroxyisobutyryl of Lys (+86.036779) if 2-hydroxyisobutyrylation peptides were enriched, and crotonyl of Lys (+68.026215) if lysine crotonylation enrichment was performed. Static carbamidomethylation of Cys (+57.0215) was set for all data sets except PXD003685 for which carbamidomethylation of Cys (+57.0215) and glutathione of Cys (+305.068156) were set as variable modifications. Falsely phosphorylated alanine was used as a decoy for phosphorylation localization analysis as described by Ramsbottom et al.^25^ For isotopically labeled samples with tandem mass tag (TMT) or SILAC, appropriate mass modifications were applied. PeptideProphet^26^ was used to assign peptide-spectrum match (PSM) probabilities on the sequence database search results. These probabilities were further refined using the iProphet tool.^27^ For acetylated and phosphorylated peptide enrichment experiments, PTMProphet^28^ was run to obtain localization probabilities.

### 2.3 PeptideAtlas Assembly

All data sets were thresholded at a probability that yields an iProphet model-based global false discovery rate (FDR) of 0.001 at the spectrum level. The exact probability threshold varied from experiment to experiment depending on how well the modeling can separate correct from incorrect information. An iProphet probability of 0.9 was used as the minimum even when global FDR analysis would favor a lower probability. Throughout the procedure, decoy identifications are retained and then used to compute the final decoy-based FDRs. The decoy-based PSM-level global FDR is 0.00017, the peptide sequence-level global FDR is 0.0008, and the protein-level global FDR is 0.007 according to the MAYU^29^ analysis.

Proteins are identified using standardized assignments to different confidence levels based on various attributes and relationships to other proteins using a 10-tier system^12^ developed over many years for the human proteome PeptideAtlas^30,31^ (Supplementary Table S1). The highest confidence level category is the “canonical” or “non-core canonical” category, which requires at least two uniquely mapping non-nested (one not inside the other) peptides at least 9 aa long with a total coverage of at least 18 aa, as required by the HPP guidelines.^32^ The decoy-based canonical protein FDR is 0.0005 with 2 decoys and 5,289 target canonical and non-core canonical proteins including contaminants.

When a group of proteins cannot be disambiguated because they contain shared peptides, one or more “leaders” of the group are categorized as “indistinguishable representative” or “representative” (Supplementary Table S2). This means that the protein or one of its close siblings is detected, but it is not possible to disambiguate them. The “marginally distinguished” category means that the protein shares peptides with a canonical entry but has some additional uniquely mapping peptide evidence that is, however, not sufficient to raise it to the canonical level. The “weak” category means that there is at least one uniquely mapping peptide that is nine or more residues long, but the evidence does not meet the criteria for being canonical. The “insufficient evidence” category means that all of the uniquely mapping peptides are less than nine residues long. Finally, all other proteins that lack any matched peptides observed above our minimum PSM significance threshold are categorized as “not observed” proteins.

## 3 Results and Discussion

The 2024 update of the *C. albicans* PeptideAtlas includes several key steps (Figure 1). Initially, suitable data sets were selected from PRIDE based on specific criteria. The MS runs from the 33 new *C. albicans* data sets^33–53^ were then organized according to protease, labeling, enrichment technique, and *C. albicans* strain used. Subsequently, these new data sets, along with one archived data set from the two preceding versions of the *C. albicans* PeptideAtlas, were reprocessed and analyzed using the TPP (version TPP v6.4.x Pillar, Build 202403120129-9139), a comprehensive end-to-end data analysis pipeline that comprises four distinct programs: PeptideProphet, iProphet, PTMProphet, and ProteinProphet. Finally, all the data was assembled taking into account the FDR employed in each program and the confidence level of the proteins identified.

**Figure 1:**
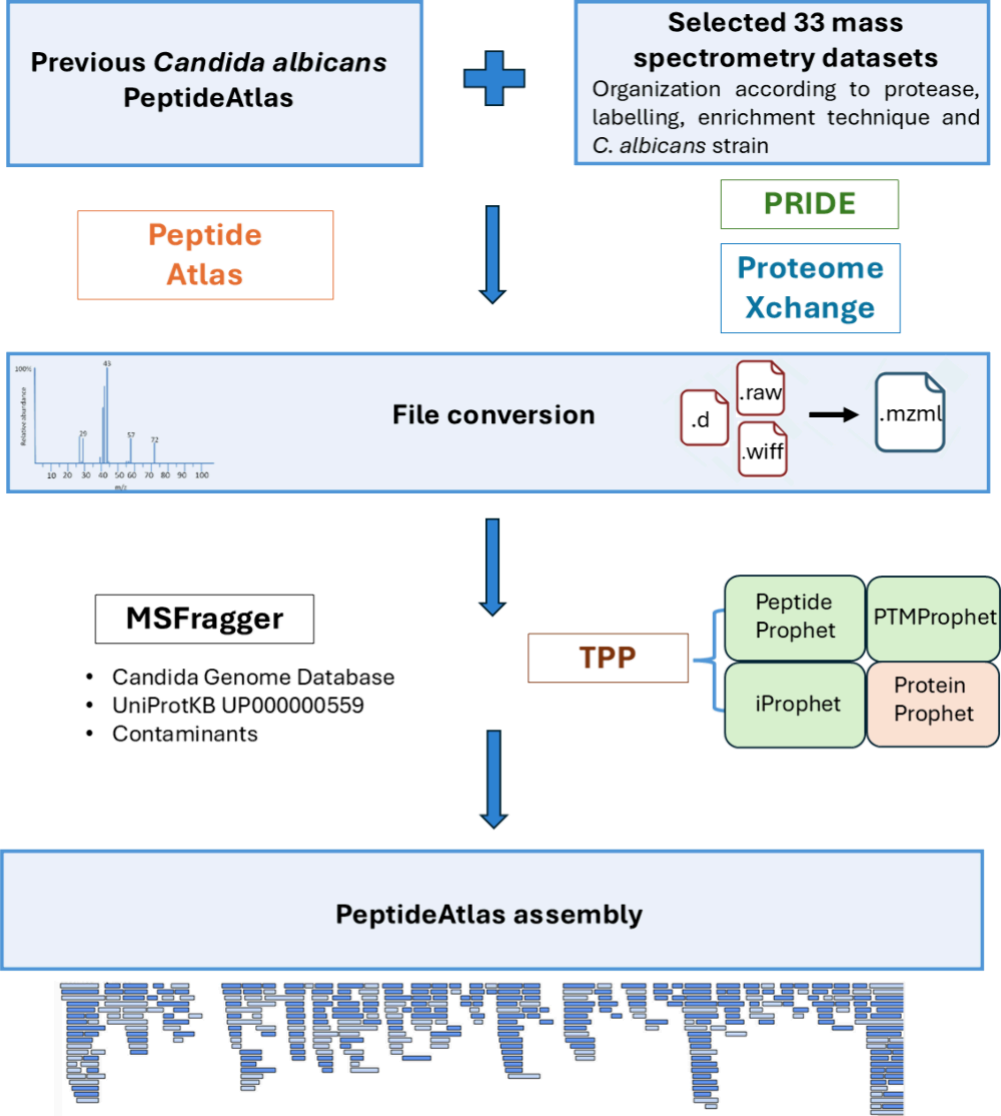
Graphical overview of the *C. albicans* PeptideAtlas upgrade. Specific steps are shown as blue boxes.

### 3.1 *C. albicans* Protein Coverage

A total of 834 MS runs (605 corresponding to the 33 data sets incorporated for the 2024 PeptideAtlas update, plus 148 from the 4 experiments added in the previous *C. albicans* PeptideAtlas build, plus 81 from the 16 experiments included in the first *C. albicans* PeptideAtlas build) generated 32,063,951 spectra of which more than one-third, 11,925,423, could be allocated to a peptide sequence. In the resulting outcome, for a decoy-based PSM FDR threshold set at 0.02%, 176,568 peptides are detected which can be explained by the minimal nonredundant set of 4,955 canonical *C. albicans* Assembly #22 protein sequences representing 79.5% of the 6,226 (as of May 2024) predicted different protein sequences.

The newly included LC-MS/MS data sets represent an increase of more than 12-fold in identified PSMs, 2.5-fold in terms of peptides, and 1.2-fold in the number of identified canonical proteins. The improvements regarding the previous and current versions of the *C. albicans* PeptideAtlas are summarized in Table 1.

**Table 1:** Summary of the Information Included in the 3 PeptideAtlas Performed to Date (2011-12, 2015-02, 2024-03) and the Improvements They Have Entailed

**PEPTIDEATLAS version**		**2011-12** ^6^	**2015-02** ^18^	**2024-03**
Data sets	Data sets incorporated	1	1	34
	Experiments analyzed	16	20	113
RUNS	Runs incorporated^a^	81	148	605
	Runs analyzed^b^	81	229	834
Spectra	New identified spectra	172,941	811,142	10,941,340
	Identified spectra	172,941	984,083	11,925,423
	Fold increase	-	5.69	12.12
Peptides	New distinct peptides	21,938	49,372	105,258
	Distinct peptides identified	21,938	71,310	176,568
	Fold increase	-	3.25	2.48
Proteins	New canonical proteins	2,563	1,553	690
	Canonical proteins identified	2,563	4,225	**4,955**
	Fold increase	-	1.65	1.17
Database	Version	A21-s02-m05-r10	A22-s05-m01-r01	A22-s07-m01-r202
	No. of proteins	6,209	6,218	6,226
Coverage	Percentage	41.3%	66.2%	79.5%
	Fold increase	-	1.60	1.20

a Number of runs included in previous versions.

b Total number of runs analyzed.

One remarkable value provided by the *C. albicans* PeptideAtlas is the report of highly confident identification of proteins corresponding to uncharacterized genes (following the terminology in CGD), i.e., those genes without previously known empirical evidence for a translated product. These amounted to 2,860 in the previous PeptideAtlas build and have notably increased to 3,180, representing 76% of the total uncharacterized genes in CGD (Figure 2). As for the verified set of genes, those that do have experimental evidence for a gene product, 87% are covered in the list of canonical proteins in this build, increasing from 1,172 identified in the previous one to 1,623. Finally, as a novelty, one dubious gene, which is unlikely to encode a product and appears indistinguishable from random noncoding sequences, was identified as a canonical protein, representing 1% of the total dubious genes.

**Figure 2:**
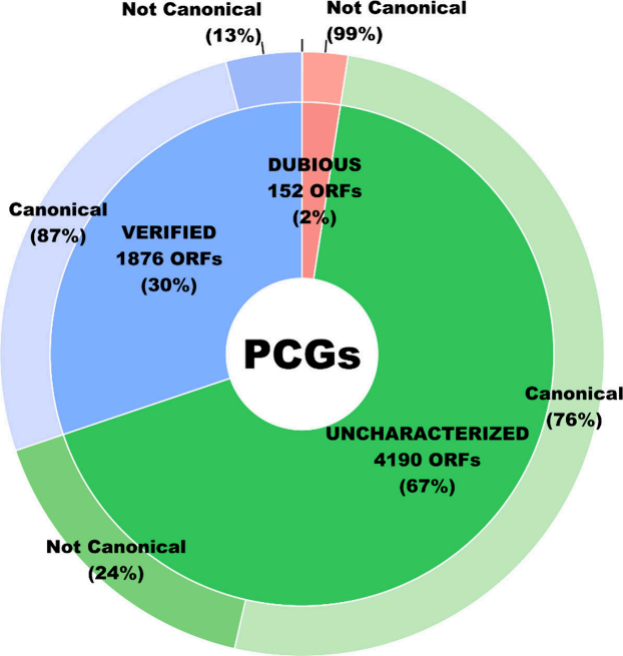
Classification of the 6,218 protein coding genes (PCGs) of *C. albicans* in Uncharacterized, Verified, and Dubious and the percentage of these covered by canonical proteins (high confidence) identified in the *C. albicans* PeptideAtlas.

An overview of the contributions of individual experiments to the entirety of PeptideAtlas is depicted in Figure 3. Figure 3A shows the cumulative identified peptides as well as distinct peptides from the 834 experiments, whereas Figure 3B shows the cumulative identified canonical proteins as well as distinct canonical proteins from the 834 experiments. In both cases, a certain region of the figure is marked for comparison in order to highlight the experiments that constituted the previous PeptideAtlas build. The build is based on 834 experiments across the 53 selected PXDs, where each PXD may be decomposed into several sub experiments. Areas in orange and red in Figure 3 indicate the total number of distinct peptides or proteins respectively in each experiment, whereas areas in blue indicate the cumulative number from the current and previous experiments.

**Figure 3:**
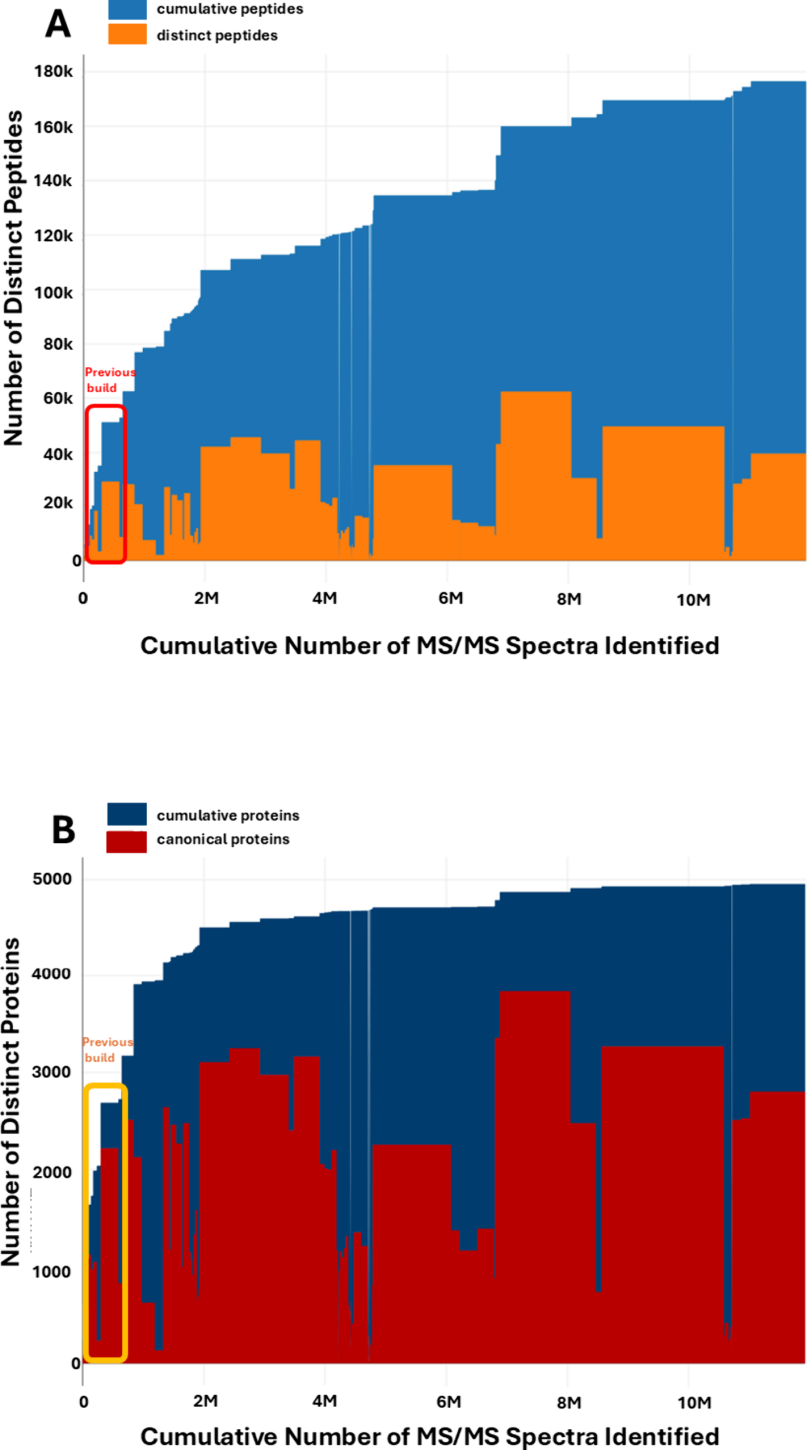
Number of distinct (nonredundant) peptides (A) and identified canonical proteins (B) as a function of the cumulative number of PSMs (peptide-spectrum matches) for the *C. albicans* PeptideAtlas update. The cumulative count is ordered by the PXD identifier (from old to new).

For a better understanding of the underlying data of this PeptideAtlas build, we calculated the frequency distributions of peptide charge state and peptide length for the approximately 12 million matched MS/MS spectra (Figure 4A). The majority of matched spectra had a charge state of 2+ (57.19%) followed by 3+ (36.42%) and 4+ (5.78%). The charge states that yield the smallest number of spectra were 5+ and 1+ with 0.12% and 0.48%, respectively. We observed a wide range of matched peptide lengths, with 7 amino acids being the shortest sequence allowed (Figure 4B). In addition, 98.8% of all matched peptides were between 7 and 37 aa long, with the most frequent peptide length of 12 aa (7.36%).

**Figure 4:**
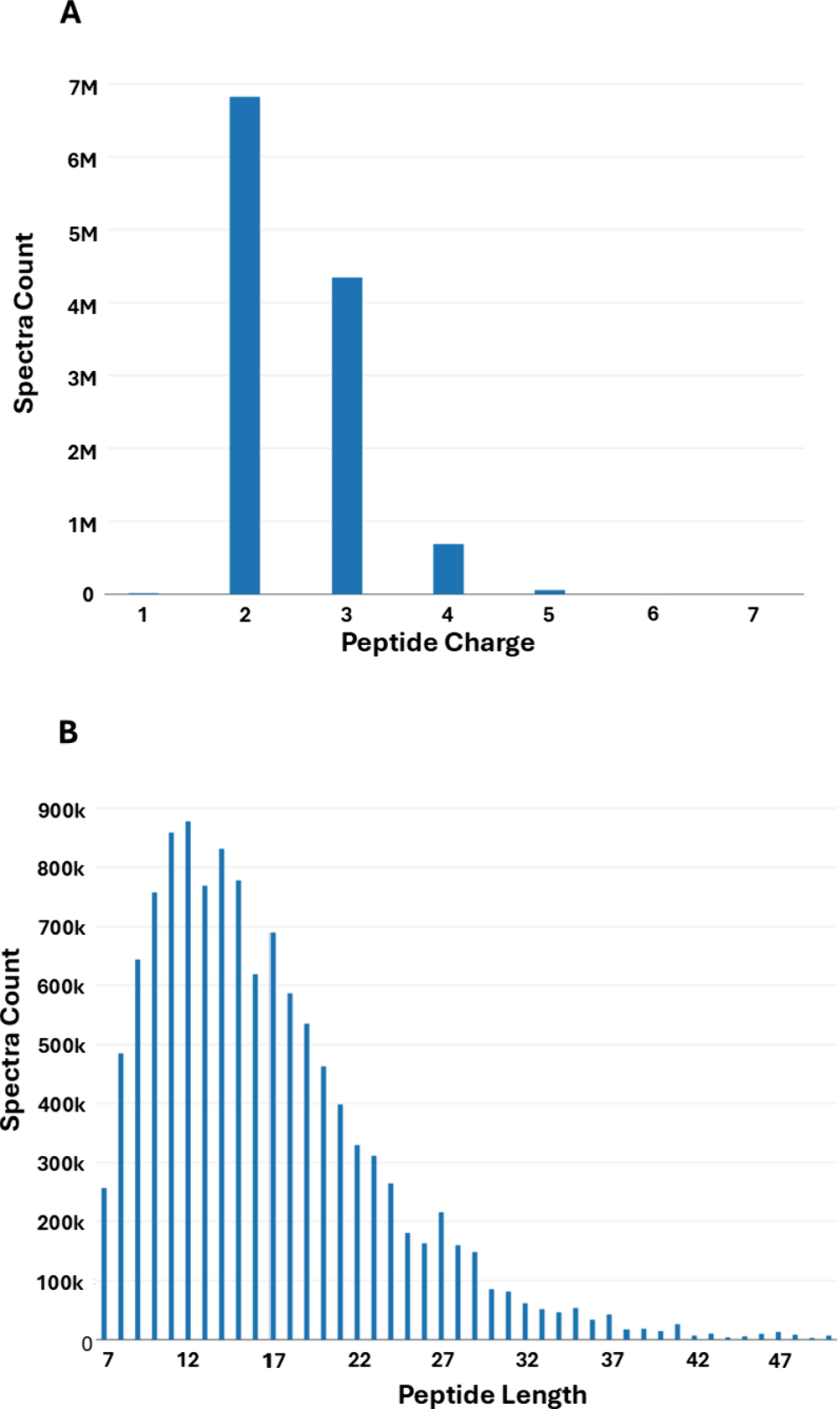
Key statistics of matched MS/MS data for the *C. albicans* PeptideAtlas. (A) Frequency distribution of the peptide charge state (*z*). (B) Frequency distribution of peptide length (aa).

At the highest level of confidence, 4,955 canonical proteins were identified in CGD (Table 2), appearing to be equally distributed along the nuclear chromosomes, with each chromosome having approximately 80% of its proteins identified. Additionally, 9 proteins were categorized as “Indistinguishable representative”, 16 as “Insufficient evidence”, 38 as “Marginally distinguished”, and 239 as “Weak” (Table 2). Finally, 956 (956/6,213 = 15.4%) predicted proteins in CGD were not identified. These “Not observed” proteins were also fairly evenly distributed across the eight nuclear chromosomes. A proteome coverage of approximately 80% represents a reasonable proportion of identified proteins. However, the remaining 20% of unidentified proteins can be attributed to several factors. One significant reason is the presence of low abundance proteins which may be below the detection limit of the instrument, making their detection challenging, physicochemical properties that do not allow their detection by the technique, proteins that are not expressed under the experimental conditions chosen or because of the nature of the primary protein sequence, in particular the absence of enzymatic cleavage sites.

**Table 2:** Proteins Identified Using CGD (A22-s07-m01-r202) Classified According to the Six PeptideAtlas Protein Categories and Their Respective Chromosomes, Including the Nuclear Chromosomes (1–7, R) and the Mitochondrial Chromosome (M)

	Entries	Canonical	%	Indistinguishable representative	%	Insufficient evidence	%	Marginally distinguished	%	Weak	%	Not observed	%
**C1**	1,383	1,144	82.7	3	0.2	4	0.3	3	0.2	47	3.4	182	13.2
**C2**	1,017	813	79.9	1	0.1	4	0.4	7	0.7	32	3.1	160	15.7
**C3**	760	588	77.4	1	0.1	2	0.3	6	0.8	44	5.8	119	15.7
**C4**	674	523	77.6	2	0.3	1	0.1	5	0.7	32	4.7	111	16.5
**C5**	523	416	79.5	0	0.0	2	0.4	3	0.6	20	3.8	82	15.7
**C6**	439	356	81.1	1	0.2	0	0.0	3	0.7	19	4.3	60	13.7
**C7**	407	323	79.4	0	0.0	0	0.0	4	1.0	11	2.7	69	17.0
**CR**	990	783	79.1	1	0.1	3	0.3	6	0.6	30	3.0	167	16.9
**CM**	20	9	45.0	0	0.0	0	0.0	1	5.0	4	20.0	6	30.0
**TOTAL**	6,213	4,955	79.8	9	0.1	16	0.3	38	0.6	239	3.8	956	15.4

To enhance proteome coverage, support for Data-Independent Acquisition (DIA) data in future versions of PeptideAtlas will be a strong point that would increase the coverage achieved. In addition, improvements in instrumentation, including the use of more sophisticated and highly sensitive mass spectrometers, will further contribute to expanding the coverage. In parallel, the use of different approaches to analyze specific subcellular fractions could improve the assessment of the proteome.

### 3.2 PeptideAtlas Tools

The *C. albicans* 2024-03 PeptideAtlas build is accessible through its web interface at https://db.systemsbiology.net/sbeams/cgi/PeptideAtlas/buildDetails?atlas_build_id=578. The general information page is displayed, which includes a “Build Overview” section containing information about the number of data sets, experiments, and MS runs, the reference database employed and the number of identified spectra, distinct peptides, and proteins classified into different protein presence levels. A “PTM coverage” section shows the number of PTM sites for phosphorylation and acetylation at several levels of confidence. Finally, it is necessary to highlight the “Experiment Contribution” and “Datasets Contribution” sections that display the contribution of each experiment and data set, respectively, for the actual *C. albicans* build in terms of spectra, peptides, and protein.

Furthermore, a linkage between CGD and PeptideAtlas ensures easy accessibility. By searching for a protein of interest in the CGD and clicking on “View peptides from PeptideAtlas” in the “Protein” section, users can access all PeptideAtlas tools. This includes viewing identified peptides in the “Sequence Motifs” section, obtaining detailed information about them either by clicking on them in this section or in the “Distinct Observed Peptides” section, assessing protein coverage in the “Sequence” section, and analyzing the probability of PTMs such as phosphorylation and acetylation within the amino acid sequence in the “PTM Summary” section. Additionally, users can see which experiments identified the protein of interest in the “Experiment Peptide Map” section. This allows observation of the expression of the 40 most highly observed peptides of the searched protein across various experiments (Figure 5).

**Figure 5:**
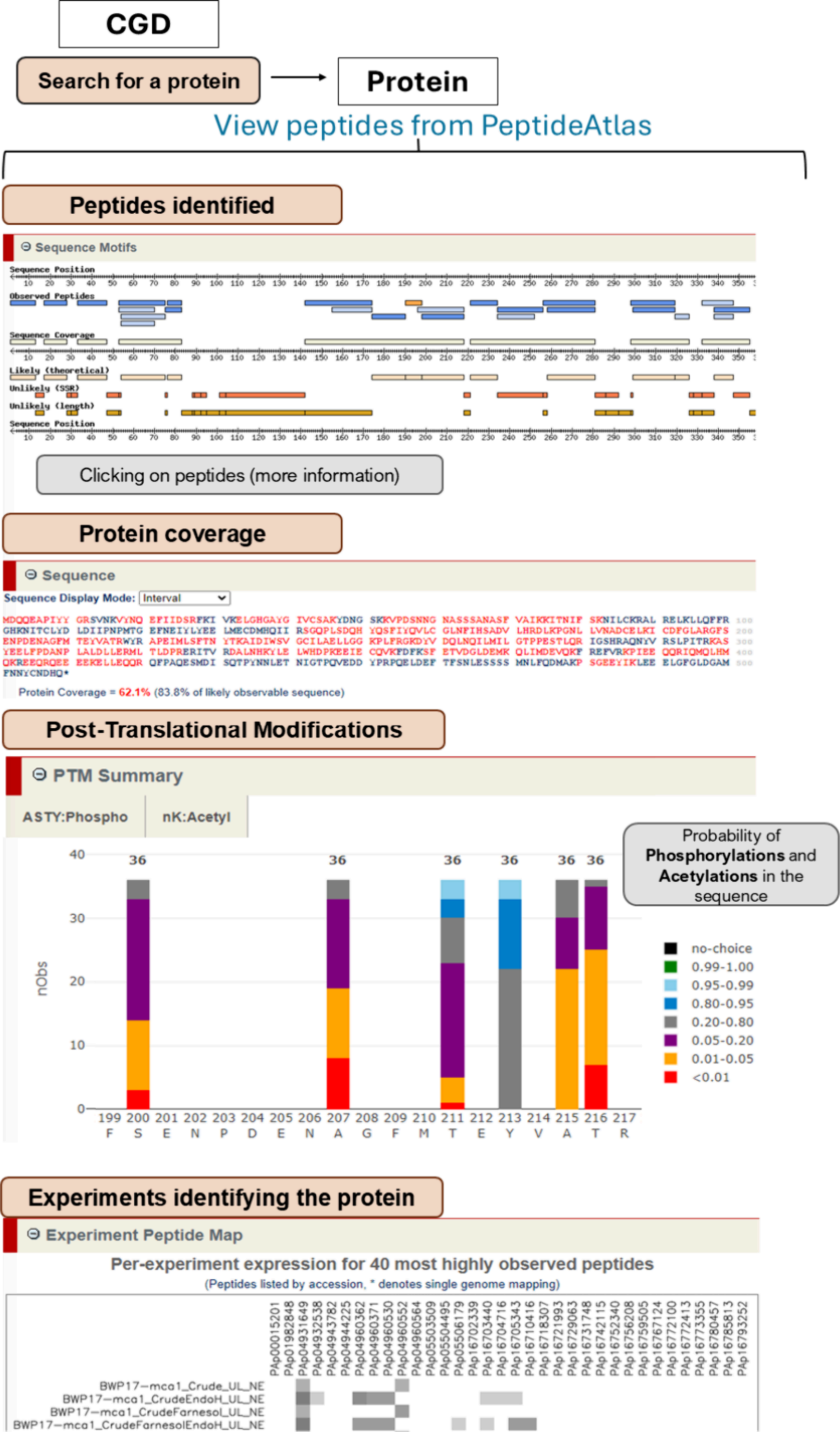
Schematic representation of the functionalities of PeptideAtlas for a given *C. albicans* protein.

The main novelty beyond the expansion of the *Candida albicans* PeptideAtlas in this update is the ability to observe the probabilities of PTMs identified with information at an individual spectral level with hyperlinked access to the annotated spectra. Consequently, the focus has been placed on providing instructions on how to interpret and apply these results.

### 3.3 Mapping Biological PTMs: Phosphorylation and Acetylation

Several PXDs that had enrichment methods for physiologically important PTMs of phosphorylation and acetylation were selected (Supplementary Table S1). A sophisticated PTM viewer in PeptideAtlas allows a detailed examination of these PTMs, including direct links to all spectral matches. Here, we illustrate two examples of canonical proteins that undergo PTMs, which are the substrate of phosphorylation and acetylation. Our summary in this publication is limited to PTMs to canonical proteins where high localization probability is appreciated, but all confidence levels of protein identification are available through the PeptideAtlas web interface. We recommend, therefore, using PeptideAtlas to evaluate specific PTM sites searched for if these are of particular interest for the reader.

Figure 6 shows the functionality of PeptideAtlas for the determination of phospho-sites. Mitogen-activated protein kinase (Mkc1) is one example in which a protein was identified as a canonical protein and for which phosphorylation has an important functional significance. The Mkc1p is part of the p42-44 MAP kinase family and is involved in the fungal cell integrity pathway, a signal transduction pathway known to be activated by cell wall stress. It has been demonstrated that Mkc1 is required for invasive hypha growth and normal biofilm development. Its activation by phosphorylation occurs after membrane perturbation, cell wall stress, and oxidative stress.^54,55^

**Figure 6:**
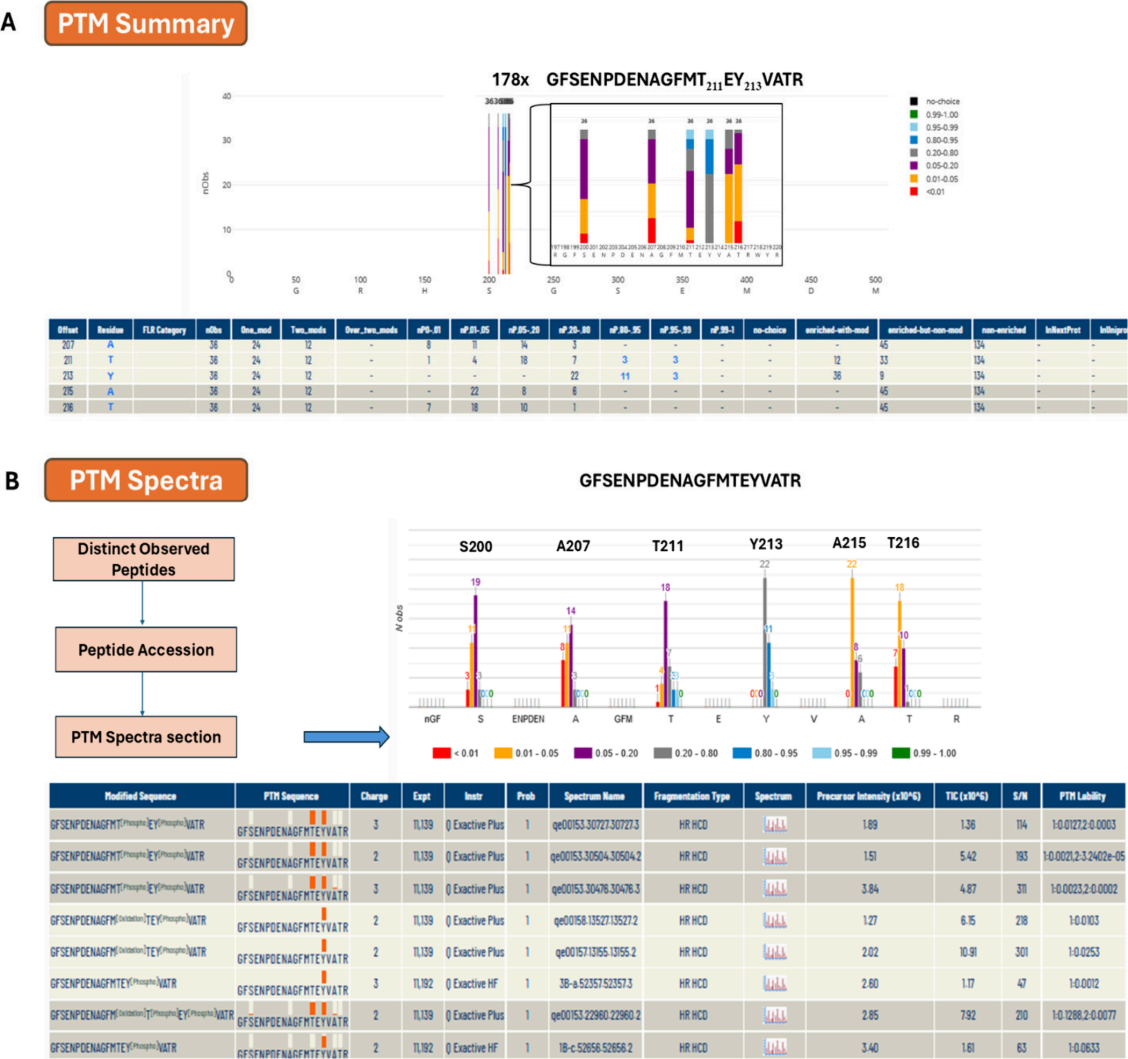
Illustration of the functionality of PeptideAtlas for the determination of phospho-sites based on the example of Mkc1 (CR_00120C_A). A) Probabilities of localization of phosphates on potential sites (as colored in bars) along the complete protein sequence. The phosphorylated residues in GFSE­NPDE­NAGF­MTEY­VATR peptide are highlighted with frequency of specific p-sites, color-coded by localization probability. This shows that the GFSE­NPDE­NAGF­MTEY­VATR peptide was observed 178 times and that T211 and Y213 were observed 36 total times each, 3 of them at the highest significance level. A numerical summary of p-site observations and information about specific sample enrichment for phospho-peptides based on metadata information collected from the individual PXD submissions is displayed below. Explanation for columns: Offset - residue offset in the protein; FLR Category - FLR punctuation; Residue - amino acid; nObs - total observed PTM spectra for the site; One_mod - the containing peptides have only one observed PTM site; Two_mods - the containing peptides have only two observed PTM sites; Over_two_mods - the containing peptides have more than two observed PTM sites; nP0-.01 - number of observed PSM with PTMProphet probability <0.01; nP.01-.05 - number of observed PSM with PTMProphet probability ≥0.01 and <0.05; nP.05-.20 - number of observed PSM with PTMProphet probability ≥0.05 and <0.2; nP.20-.80 - number of observed PSM with PTMProphet probability ≥0.2 and <0.8; nP.80-.95 - number of observed PSM with PTMProphet probability ≥0.80 and <0.95; nP.95-.99 - number of observed PSM with PTMProphet probability ≥0.95 and <0.99; nP.99-1 - PTMProphet probability ≥0.99; no choice - number of PSMs covering this site for which there was no choice in the localization of the PTM. Only one residue was an S, T, or Y; enriched-with-mod - number of PSMs covering this site with phospho modification on this site, and originating from a phospho-enriched sample; enriched-but-nonmod - number of PSMs covering this site with no phospho modification anywhere on the peptide but yet originating from a phospho-enriched sample; nonenriched - number of PSMs covering this site with no phospho modification anywhere on the peptide and originating from a nonenriched sample, typically not searched for phospho; inNexProt - PTM site annotated in neXtprot; InUniProt - PTM site annotated in UniProt. B) Detailed view of the phosphorylated peptide GFSE­NPDE­NAGF­MTEY­VATR and phospho-site localization probability distributions. The lower panel shows information at an individual spectral level with hyperlinked access to the annotated spectra.

Mkc1 (CR_00120C_A) was identified with 34 distinct peptides and 62.1% protein sequence coverage. The *C. albicans* PeptideAtlas shows only one phosphorylated peptide: GFSE­NPDE­NAGF­MTEY­VATR identified 36 times. In 3 cases, both of the phosphorylation sites at positions T211 and Y213 were identified with a significance of 0.95 < *P* < 0.99 in light blue with additional observations for these sites at lower probabilities. These are serine S200 and threonine T216 whose phosphorylation sites were assigned with low probabilities, indicating with high confidence that the detected phosphate was not positioned at those sites.

In Figure 6A a numerical summary of the phosphorylation sites is displayed. Peptide GFSE­NPDE­NAGF­MTEY­VATR was detected 134 times in samples that were not enriched in phosphorylated peptides, 12 times as a single phosphorylated peptide, and 24 times as a doubly phosphorylated peptide from enriched samples. T211 was identified exclusively in the doubly phosphorylated peptide. Y213 was identified in all 36 observations. Figure 6B shows a detailed peptide view of the phosphorylated peptide GFSE­NPDE­NAGF­MTEY­VATR and phospho-site identification scores. The lower panel in Figure 6B shows information at the individual spectral level with hyperlinked access to the annotated spectra. Together, this strongly suggests that Mkc1 is phosphorylated at T211 and Y213. Navarro-García et al.^56^ established that phosphorylation of the threonine and tyrosine residues in the TEY signature of domain VIII of the p42-44 MAP kinase family is necessary for phosphorylation activity. When both residues are dephosphorylated, p42-44 MAP kinases are unable to phosphorylate their substrates.

Figure 7 demonstrates the functionality of PeptideAtlas for the determination of acetylated residues. Hsp90 (C7_02030W_A) is one example in which a protein was identified as a canonical protein and for which acetylation was previously demonstrated to have an important functional significance. Hsp90 is a conserved molecular chaperone that facilitates the folding and function of hundreds of proteins, many of which serve as core hubs of signal transduction networks. Hsp90 governs morphogenesis and virulence such that a compromise of Hsp90 function induces the transition from yeast to filamentous growth in the absence of any additional inducing cue. Hsp90 governs temperature-dependent morphogenesis by inhibiting signaling through the cAMP-protein kinase A (PKA) pathway.^57^ Furthermore, it is necessary to highlight the important role of Hsp90 in mediating echinocandin and biofilm azole resistance.^58^

**Figure 7:**
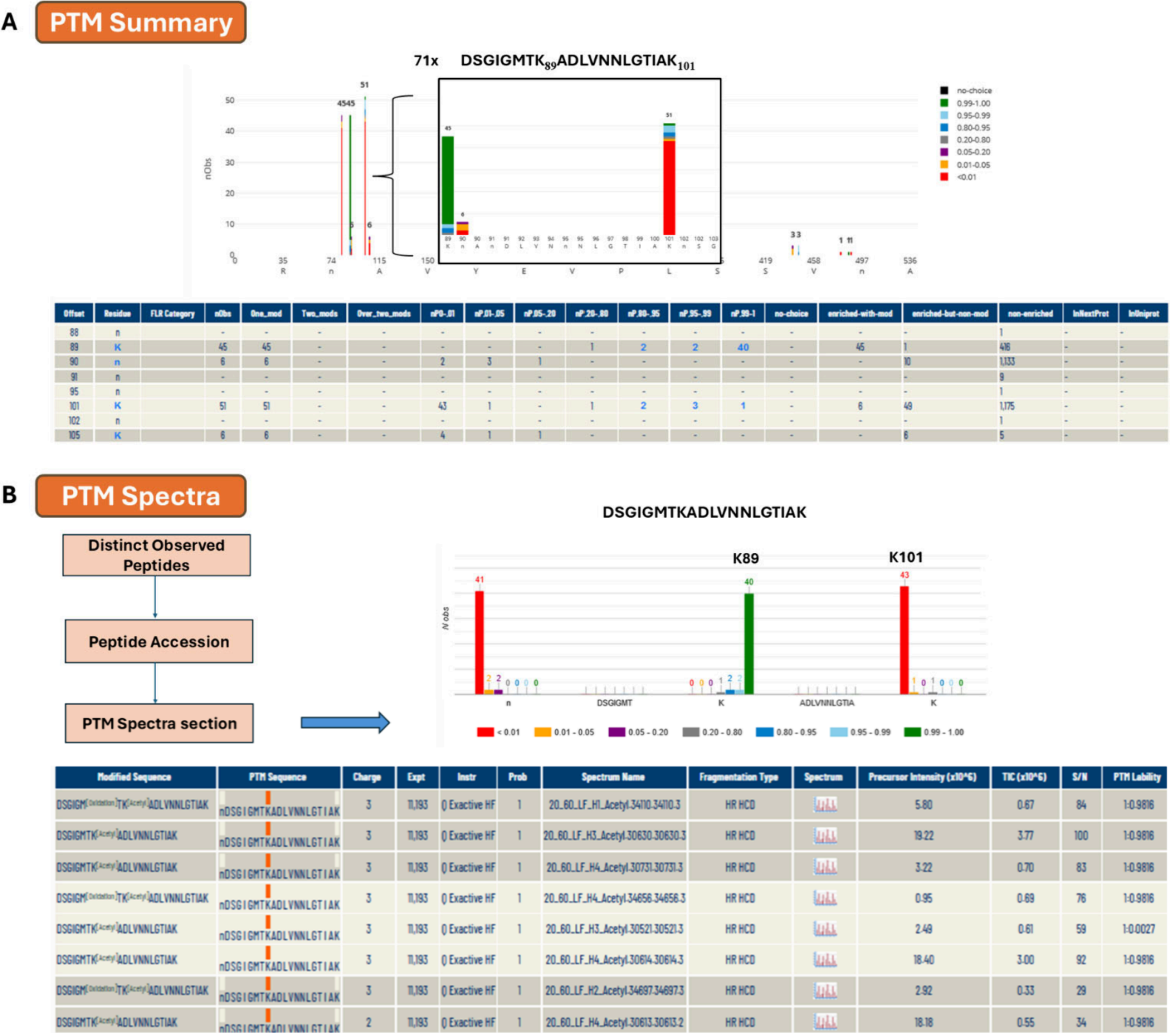
Illustration of the functionality of PeptideAtlas for the determination of acetylated residues based on the example of Hsp90 (C7_02030W_A). A) Probabilities of localization of acetylated sites in DSGI­GMTK­ADLV­NNLG­TIAK peptide are color-coded by localization probability. This shows that the acetylated peptide was observed 71 times and that K89 and K101 were observed 45 and 51 total times, respectively. A numerical summary of acetylated site observations and information about specific sample enrichment for acetylated peptides based on metadata information collected from each individual PXD submission is displayed below. Explanation for columns: Offset - residue offset in the protein; FLR Category - FLR punctuation; Residue - amino acid; nObs - total observed PTM spectra for the site; One_mod - the containing peptides have only one observed PTM site; Two_mods - the containing peptides have only two observed PTM sites; Over_two_mods - the containing peptides have more than two observed PTM sites; nP0-.01 - number of observed PSM with PTMProphet probability <0.01; nP.01-.05 - number of observed PSM with PTMProphet probability ≥0.01 and <0.05; nP.05-.20 - number of observed PSM with PTMProphet probability ≥0.05 and <0.2; nP.20-.80 - number of observed PSM with PTMProphet probability ≥0.2 and <0.8; nP.80-.95 - number of observed PSM with PTMProphet probability ≥0.80 and <0.95; nP.95-.99 - number of observed PSM with PTMProphet probability ≥0.95 and <0.99; nP.99-1 - PTMProphet probability ≥0.99; no choice - number of PSMs covering this site for which there was no choice in the localization of the PTM (only one residue was an S, T, or Y); enriched-with-mod - number of PSMs covering this site with phospho modification on this site and originating from a phospho-enriched sample; enriched-but-nonmod - number of PSMs covering this site with no acetyl modification anywhere on the peptide anywhere on the peptide but yet originating from a acetyl-enriched sample; nonenriched - number of PSMs covering this site with no acetyl modification anywhere on the peptide and originating from a nonenriched sample, typically not searched for acetylation; inNexProt - PTM site annotated in neXtprot; InUniprot - PTM site annotated in UniProt. B) Detailed view of the acetylated peptide DSGI­GMTK­ADLV­NNLG­TIAK and acetylation site localization probability distributions. The lower panel shows information at an individual spectral level with hyperlinked access to the annotated spectra.

Hsp90 (C7_02030W_A) was identified with 355 peptides and 96.1% protein sequence coverage. In Figure 7 we present the acetylated peptide DSGI­GMTK­ADLV­NNLG­TIAK identified 71 times. In 40 cases the acetylated K89 was identified with a significance of 0.99 < *P* < 1.00 with additional observations for this site at lower probability. There is another acetylation site at K101 assigned with low probability, indicating high confidence that the detected acetyl was not positioned at this site.

In Figure 7A a numerical summary of the acetylation sites is displayed. K89 was observed 416 times in samples that were not enriched for acetylated peptides, 45 times in a single acetylated peptide from enriched samples, and 51 times for K101 in an acetylated peptide from enriched samples.

There are several peptides of C7_02030W_A where acetylation was observed, such as peptide LFLK­EDQL­EYLE­EKR which was identified 1022 times. In 3 cases the acetylated K191 was identified with a significance of 0.99 < *P* < 1.00 with additional observations for this site at lower probability. The peptide VVVS­YKLV­DAPA­AIR was identified 8 times; for 3 of them, the acetylated K567 was identified with a significance of 0.99 < *P* < 1.00 with additional observations for this site at lower probability.

## 4 Conclusion

The 2024 update of the *C. albicans* PeptideAtlas (2024-03 version) includes 33 new data sets chosen from PRIDE based on specific criteria (such as the use of SC5314 or mutants, LC-MS/MS, DDA mode, nonglycosylated enrichment, and CGD) to now include extensive identification of PTMs. Finally, these experiments were reanalyzed in conjunction with data from previous versions of PeptideAtlas using the TPP.

The 2024 *C. albicans* PeptideAtlas build resulted in a 12-fold increase in the number of identified PSMs, a 2.5-fold increase in characterized peptides, and a 1.2-fold increase in proteins compared to the previous build. A total of 11,925,423 PSM, 176,568 peptides, and 4,955 canonical protein sequences make it the most comprehensive proteomics resource available up to date with a coverage of 80% of the total predicted proteome.

In addition, highly confident protein identifications have been made for 76% of the genes labeled as “uncharacterized” (without a known protein product) and 1% of the genes categorized as “dubious” (unlikely to encode a product) in CGD. A total of 3,382 phosphorylated serine and 536 phosphorylated threonine sites were identified, with false localization rates of 1% and 5.3% and PTMProphet scores ranging between 0.95 and 0.99, respectively. The majority of spectra matched to peptides have a charge state of 2+ (57.19%), and the most common peptide length is 12 aa (7.36%).

Approximately 80% of the proteins belonging to nuclear chromosomes were identified as canonical proteins, and 15% of them were not observed.

An explanation of the PeptideAtlas tools and instructions on how to access them, either through the PeptideAtlas web interface or via the CGD, have been provided. Accessing PeptideAtlas via its own web interface offers information on the “Build overview”, “PTM coverage”, “Experiment contribution”, and “Data set contribution”. Through a protein entry on the CGD Web site, users can view information about the peptides identified for that protein, protein coverage, probability of phosphorylation and acetylation within the sequence, and the experiments in which the protein has been identified. A detailed explanation of how to interpret and apply PTM detection and localization information was provided. This latest expanded *C. albicans* PeptideAtlas build provides a comprehensive compendium of results from community proteomics data that will enable further research on this important pathogen.

## Supplementary Material





## Data Availability

The mass spectrometry proteomics data used for the PeptideAtlas update is deposited at the ProteomeXchange Consortium via the PRIDE partner repository whose data set identifiers are listed in Supplementary Table S1.

## Notes

Supplementary Table S1: Summary information on the 33 selected PXD data sets for the PeptideAtlas update (XLSX)
Supplementary Figure S1: Accumulative PXDs (PRIDE) with verified *C. albicans* content by year (2015-03/2024-04); Supplementary Table S2: Technical definition of protein identification confidence categories in the *C. albicans* PeptideAtlas build (PDF)

## Abbreviations

CGD: *Candida* Genome Database
DDA: Data-dependent analysis
FDR: False discovery rate
LC-MS/MS: Liquid chromatography tandem mass spectrometry
PRIDE: Proteomics identification database
PSM: Peptide spectrum match
PTM: Post-translational modifications
TMT: Tandem mass tag
TPP: Trans-proteomic pipeline
