# Supplementary material for: Candida albicans: A Comprehensive View of the Proteome
Source: J Proteome Res. 2025 Mar 14;24(4):1636–48. doi: 10.1021/acs.jproteome.4c01020 (PMC12123666; doi:10.1021/acs.jproteome.4c01020)
Supplement: Supplementary file 2 [file pr4c01020_si_002.pdf]

Candida albicans: **a comprehensive view of the proteome**

Leticia Gomez-Artiguez[1#], Samuel de la Cámara-Fuentes[2#], Zhi Sun[3#], María Luisa Hernáez[2], Ana Borrajo[1], Aída Pitarch[1], Gloria Molero[1], Lucía Monteoliva[1], Robert L. Moritz [3], Eric W. Deutsch[3] and Concha Gil[1,2*]

[1] Microbiology and Parasitology Department, Faculty of Pharmacy, Complutense University of Madrid, 28040 Madrid.; [2] Proteomics Unit, Faculty of Pharmacy, Complutense University of Madrid, 28040 Madrid.; [3] Institute for Systems Biology, 401 Terry Ave North, Seattle, WA, USA. 98109.

# Leticia Gomez-Artiguez, Samuel de la Cámara-Fuentes and Zhi Sun contributed equally to this work.

*Corresponding author: Concha Gil: conchagil@ucm.es

Supporting Information

Supporting Information is available free of charge at the JPR web site.

Supplementary Table S1. Summary information on the 33 selected PXD datasets for the PeptideAtlas update. (xlsx)

Supplementary Figure S1. Accumulative PXDs (PRIDE) with verified *C. albicans* content by year (2015-03/2024-04).

Supplementary Table S2. Technical definition of protein identification confidence categories in the *C. albicans* PeptideAtlas build.

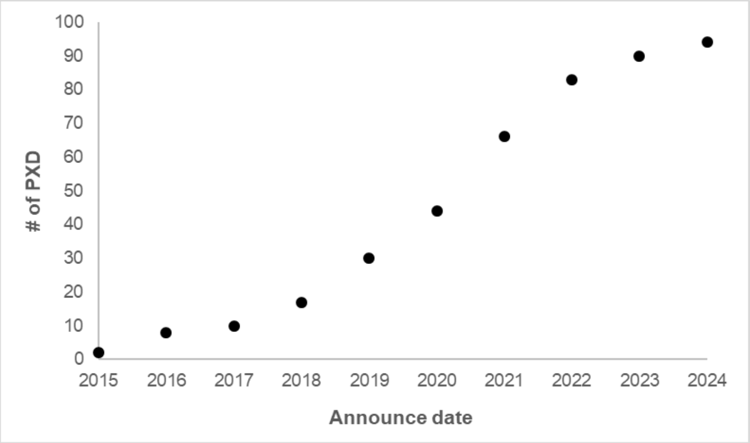

Supplementary Figure S1. Accumulative PXDs (PRIDE) with verified *C. albicans* content by year (2015-03/2024-04).

Supplementary Table S2. Technical definition of protein identification confidence categories in the *C. albicans* PeptideAtlas build.

Protein label	Technical definition
Canonical	Proteins with at least two 9AA or greater peptides with a total extent of 18AA or greater that are uniquely mapping within the core reference proteome.
Noncore-Canonical	Proteins with at least 9AA or greater peptides with a total extent of 18AA or greater that do not map in the core reference proteome, but rather to an isoform, contaminant, or other protein missing from the core reference proteome.
Indistinguishable Representative	Protein has no unique peptides, and there are several indistinguishable proteins. The former is assigned to be an Indistinguishable Representative, while the latter are Indistinguishable
Insufficient Evidence	Protein has more unique peptides than shared peptides, but none are 9AA or greater.

Marginally Distinguished	Protein has unique peptides, but there are not more unique peptides than shared peptides, and the extended length of unique peptide is < 18AA.
Weak	Protein has more unique peptides than shared peptides, and only one uniquely mapping peptide 9AA or greater.
Not observed	Protein has no peptides above our PSM significance threshold. It may have PSMs of low significance, but these are not considered.
